# InCURA: integrative gene clustering based on transcription factor binding sites

**DOI:** 10.1093/nar/gkaf1377

**Published:** 2025-12-19

**Authors:** Lorna Rinck, Ricardo O Ramirez Flores, Julio Saez-Rodriguez, Mahak Singhal

**Affiliations:** European Center for Angioscience (ECAS), Medical Faculty Mannheim, Heidelberg University, 68167 Mannheim, Germany; Institute for Computational Biomedicine, Faculty of Medicine and Heidelberg University Hospital, Heidelberg University, 69120 Heidelberg, Germany; European Bioinformatics Institute, European Molecular Biology Laboratory, Hinxton, Cambridgeshire, CB10 1SD, United Kingdom; Institute for Computational Biomedicine, Faculty of Medicine and Heidelberg University Hospital, Heidelberg University, 69120 Heidelberg, Germany; European Bioinformatics Institute, European Molecular Biology Laboratory, Hinxton, Cambridgeshire, CB10 1SD, United Kingdom; European Center for Angioscience (ECAS), Medical Faculty Mannheim, Heidelberg University, 68167 Mannheim, Germany; Helmholtz-Institute for Translational AngioCardioScience (HI-TAC) of the Max Delbrück Center for Molecular Medicine in the Helmholtz Association at Heidelberg University, 69117 Heidelberg, Germany

## Abstract

Biologically meaningful interpretation of transcriptomic datasets remains challenging, particularly when context-specific gene sets are either unavailable or too generic to capture the underlying biology. We here present InCURA, an integrative clustering strategy based on transcription factor (TF) motif occurrence patterns in gene promoters. InCURA takes as input lists of (i) all expressed genes, used solely to identify dataset-specific expressed TFs, and (ii) differentially regulated genes (DRGs) used for clustering. Promoter sequences of DRGs are scanned for TF binding motifs, and the resulting counts are compiled into a gene-by-TFBS matrix. InCURA then uses unsupervised clustering to infer gene modules with shared predicted regulatory input. Applying InCURA to diverse biological datasets, we uncovered functionally coherent gene modules revealing upstream regulators and regulatory programs that standard enrichment or co-expression analyses fail to detect. In summary, InCURA provides a user-friendly, regulation-centric tool for dissecting transcriptional responses, particularly in settings lacking context-specific gene sets.

## Graphical Abstract

**Graphical Abstract:**
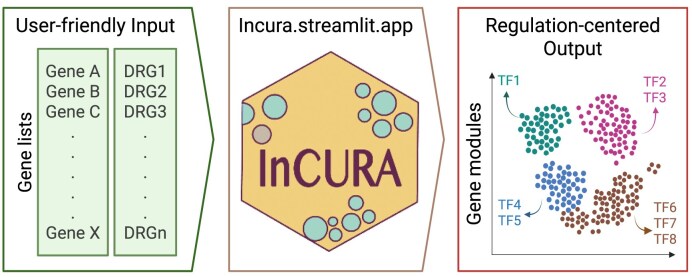

## Introduction

High-throughput transcriptomic profiling has become a cornerstone of modern life science research. Understanding how gene expression is regulated across different contexts is central to uncovering mechanisms that drive cellular function in development, homeostasis, and disease [1, 2]. Therefore, the identification of differentially regulated genes (DRGs), such as differentially expressed genes (DEGs) or differentially accessible regions (DARs) linked to genes, across conditions, tissues, and time points has become a routine analysis of transcriptomic and epigenomic datasets, yet it is only the first step toward understanding the underlying regulatory mechanisms that shape these changes. A list of genes alone provides limited biological insight unless it can be contextualized in terms of functional pathways or regulatory control. Therefore, for drawing biological interpretations, researchers frequently employ gene set enrichment analysis [3] or over-representation analysis using curated gene set catalogues from databases like the Gene Ontology (GO) [4], WikiPathways [5], KEGG [6], or Reactome [7]. These resources, while valuable, are often too generic, not tailored to specific experimental, tissue, or cell-type contexts, or biased to certain research areas [8, 9]. This is particularly problematic in dynamic or poorly annotated systems, where predefined gene sets may miss subtle or novel regulatory programs [10]. As a result, the existing toolbox is missing broadly applicable approaches that help dissect datasets and enable the extraction of truly biologically meaningful insights.

Addressing the limitations of the classical enrichment approach of predefined gene sets, co-expression-based methods such as weighted gene co-expression network analysis (WGCNA) or non-negative matrix factorization were established for identifying gene modules with coordinated expression patterns [11, 12]. These approaches can uncover gene communities potentially involved in related biological processes and are particularly useful when prior knowledge is limited [13]. However, co-expression clustering relies exclusively on correlation in gene expression levels, which may not directly reflect shared TF–target gene interactions of two or more co-expressed genes [14, 15]. Moreover, most implementations consider the entire expressed transcriptome, which can introduce substantial noise from genes unrelated to the condition of interest [16]. As a result, subtle regulatory relationships among smaller, condition-specific subsets of genes can be obscured, limiting the biological resolution of these analyses [17, 18].

While co-expression can suggest functional connections, it only indirectly informs about regulatory control. Transcription factors (TFs) play a central role in regulating gene expression by binding to specific sequence motifs in DNA [19, 20]. Notably, TF binding sites (TFBS) are known to accumulate densely in regions proximal to the transcription start site (TSS) [21], making promoter regions a rich and functionally relevant source of regulatory information. Computational scanning of promoter sequences for TFBS, using tools such as FIMO from the MEME Suite, enables the prediction of potential regulatory inputs for individual genes [22, 23]. Importantly, genes that share similar combinations of TFBS in their promoters are likely to be under coordinated regulatory control, even if the magnitude of their expression is not strongly correlated [24]. Thus, regulatory motif analysis provides a complementary and mechanistically grounded perspective for identifying gene modules shaped by shared transcriptional regulation [25, 26]. Related approaches, such as TF activity estimation, often fall under the broader umbrella of enrichment analysis and aim to score TFs based on the expression of their known target genes [27, 28]. While these methods are effective for nominating individual regulators, they typically do not resolve gene modules or capture the combinatorial action of multiple TFs acting together on gene sets. As such, promoter-based clustering strategies offer a valuable extension to this class of methods by directly linking genes through shared predicted regulatory input.

To overcome the limitations of expression-based clustering and incorporate regulatory information directly into the analysis, we developed InCURA, an integrative gene clustering based on TFBS occurrences. InCURA focuses specifically on gene-level features and integrates TFBS predictions to identify clusters of genes with shared upstream regulatory profiles, making it independent of predefined gene sets. By prioritizing promoter-level information and limiting analysis to condition-relevant genes, InCURA was designed to extract interpretable, biologically meaningful gene modules in a context-specific manner. We demonstrate that InCURA identifies functionally coherent gene modules and highlights upstream regulators that are consistent with known biology. Through case studies, we showcase that InCURA captures regulatory relationships that would otherwise be missed by one of the most commonly used co-expression-based methods, WGCNA. Together, our results position InCURA as a valuable tool for dissecting transcriptional programs in a regulation-centered, context-aware manner, especially in the absence of curated gene sets.

## Methods

### Data acquisition and preprocessing

All data processing and analyses were conducted using Python v3.10 and R v4.3.3 packages. Apptainer (Singularity) image definition files and singularity image files are provided on Zenodo, ensuring full reproducibility of the computational environment.

### Bulk RNA-seq dataset (Case study 1 and 2)

The bulk RNA-seq dataset used for the first case study was obtained from a previously published study on T cell exhaustion in a TOX knockout model [29]. In this dataset, CD8⁺ T cells were isolated from wild-type and TOX-deficient mice, and transcriptomic profiling was performed to assess differential gene expression associated with the loss of TOX, a key regulator of T cell exhaustion. Raw RNA count files were downloaded from the Gene Expression Omnibus (GEO) under accession number GSE132987. The data were imported into a Python environment and structured using the anndata format for downstream analysis. Quality control was performed using scanpy *v1.11.0* and the decoupler *v2.1.1* package with default parameters [28, 30]. Differential expression analysis (DEA) between TOX knockout and wild-type samples was carried out using pyDESeq2 *v0.5.0*, a Python implementation of the DESeq2 algorithm [31, 32]. Genes with an adjusted *P*-value ≤ .05 were considered significantly differentially expressed. These DEGs were extracted and saved as a plain-text file to be used as input for the InCURA workflow. The gene signatures used for functional evaluation of the resulting clusters were curated based on marker genes defined by the original authors in their study (Supplementary File). The bulk RNA-seq dataset of the second case study was based on a dataset investigating B cell subsets in systemic lupus erythematosus (SLE). The raw counts and metadata were downloaded from GEO under the accession number GSE110999 [33]. The same analytical workflow was applied as for the T cell dataset: quality control using decoupler and DEA using pyDESeq2. The dataset was subsetted to include only samples from SLE patients and healthy controls, excluding rheumatoid arthritis patients. We performed two separate contrasts, CD11chi B cells versus memory B cells and CD11chi B cells versus naive B cells, and extracted the union of significantly DEGs across both comparisons, using an adjusted *P*-value threshold of 0.05. This union set of DEGs was then submitted to the InCURA workflow. For the functional evaluation of resulting clusters, we used a marker gene signature for CD11chi B cells derived from the supplementary material of the original study. Specifically, we selected genes from the first eight marker gene categories (CD11chi Phenotype, Activation, BCR signaling, Cytokines/Receptors, Differentiation), retaining only those with a log₂ fold change >10 in both contrasts: CD11chi versus Naive B cells as well as CD11chi versus Memory B cells (Supplementary File).

### Single-cell RNA-seq dataset (Case study 3)

The second case study was based on a publicly available single-cell RNA-seq dataset of FACS-sorted hepatocytes from adult mice with either a hepatocyte-specific double knockout of the core circadian regulators REV-ERBα and REV-ERBβ or a wild-type genotype. We downloaded the preprocessed Seurat object from the GEO under the accession number GSE143528 [34]. From the full dataset, we subsetted the data to retain only hepatocytes. Cells originating from different liver lobule zones were then merged into a single zone-unaware hepatocyte population to increase statistical power and focus the analysis on genotype-driven effects rather than spatial heterogeneity. DEA between double knockout and wild-type hepatocytes was performed using Seurat’s *v4.3.1* FindMarkers function with default settings [35, 36]. We used the adjusted *P*-value to assess statistical significance and applied a threshold of adjusted *P* ≤ .05 to identify DEGs. The resulting DEG list was then extracted and used as the input for InCURA.

### Database-derived differentially expressed genes (Case study 4)

For the third case study, we used a web-based resource associated with a single-cell RNA-seq study of mouse gastrulation, in which individual cells were computationally grouped into metacells and assigned embryonic age estimates by the original authors (https://apps.tanaylab.com/MCV/embflow/) [37]. The accompanying web tool provides multiple functionalities, including on-demand DEA between any two metacells, with results displayed as tables containing gene names and associated statistical metrics. To investigate developmental transitions within the mesoderm lineage, we selected three metacells (IDs: 329, 84, and 154) representing early, intermediate, and late stages along the mesodermal trajectory. Based on the authors’ annotations and literature references, these metacells correspond to primitive streak, early nascent mesoderm, and rostral mesoderm cell states, respectively. We performed DEA between the early and intermediate metacells, and separately between the intermediate and late metacells, using the built-in functionality of the web tool. The resulting gene lists were manually extracted from the output tables and saved as plain-text files for use as input in the InCURA workflow. The cell state-specific gene signatures used for functional evaluation of the resulting clusters were curated based on marker genes defined by the original authors in their study (Supplementary File).

### Features derived from chromatin accessibility data

To demonstrate the application of InCURA to non-transcriptomic features, we extracted genes linked to DARs from the supplementary material of the original study [38]. For these genes, promoter regions were scanned using the full set of human TFs to quantify TF binding motif occurrences as input for the InCURA analysis.

### TF motif query

To identify TFBS in promoter regions, we first filtered the list of expressed genes for TFs. As a reference set of TFs, we used curated lists of known mouse and human TFs provided by the cisTarget resource [39, 40]. The corresponding TF motifs were retrieved using the MotifDB R package [41], which aggregates position weight matrices (PWMs) from multiple publicly available motif databases [29]. The selected motifs were exported in MEME format, which is compatible with downstream motif scanning tools, such as FIMO from the MEME Suite [22, 23].

### Promoter definition and TFBS scanning

Promoter regions were defined as the genomic intervals spanning –2000 to +500 base pairs relative to the TSS. Promoter sequences were extracted using betools *v2.27.1* and the Python package promoterExtract (https://pypi.org/project/promoterExtract/), based on Ensembl genome assemblies (mm10 for mouse and hg38 for human). The extracted sequences were sorted by Ensembl gene ID and annotated with corresponding gene symbols for downstream matching. Next, promoter sequences were filtered to retain only those corresponding to the input list of DRGs. The filtered set of promoters was then scanned for TF binding motifs using FIMO from the MEME Suite *v5.5.7*. Only motifs associated with the list of expressed TFs were included in the query. FIMO was run using a Markov background model generated from the input promoter sequences using FIMO’s native background function. An FDR threshold of 10% was applied to identify significant motif hits.

### Downstream processing of motif hits

Following motif scanning, overlapping motif hits for the same TF within the promoter region of the same gene (DRGs) were consolidated into single entries. Specifically, multiple occurrences of the same motif with overlapping genomic coordinates (minimum 1 bp overlap) were collapsed to avoid redundant counting of densely clustered binding sites. A motif count matrix was then constructed by tallying the number of non-overlapping motif occurrences for each TF in the promoter region of each DEG, resulting in a matrix of dimensions *n*_DRGs_× *n*_TF_. This matrix was then used as the input for *k*-means clustering to group genes into transcriptionally coherent modules based on their shared TFBS architecture. Genes are clustered without separating up- and down-regulated genes, allowing the workflow to unbiasedly capture shared regulatory patterns. Directional effects can be examined within clusters downstream if desired. Cluster assignments were saved in tab-separated value files for downstream analysis. The number of clusters (*k*) was determined by a combination of visual inspection of cluster compactness and silhouette score analysis using sklearn *v1.5.2* and scipy *v1.15.2*, selecting the highest *k* value before a pronounced decline in silhouette scores was observed, balancing resolution with stability. To visualize the cluster structure, the dimensionality of the TFBS count matrix was reduced using UMAP (from umap *v0.5.7)* with Canberra distance (*n_neighbors* = 15, *min_dist* = 0.2) and the cluster labels from the *k*-means clustering.

### Transcription factor prioritization

To identify putative regulators driving each gene cluster, TFs were ranked based on their motif enrichment within the promoters of clustered genes. For this purpose, the motif count matrix was first binarized to indicate the presence or absence of each TF binding motif in the promoter of each gene. For each cluster, a Fisher’s exact test was performed to assess whether motifs for a given TF were significantly enriched in the promoters of genes within the cluster compared to the background set, comprising all genes from the remaining clusters. *P*-value adjustment was performed using the Benjamini–Hochberg method, and a threshold of 0.05 was applied to identify significantly enriched TFs. To focus on cluster-specific regulatory drivers, ubiquitously enriched TFs, those significantly enriched across multiple clusters, were removed. This filtering step ensured that only uniquely enriched TFs were retained for downstream interpretation as candidate regulators specific to individual gene modules.

### Functional enrichment analysis

To evaluate the biological relevance of the identified gene clusters, we conducted two complementary types of enrichment analysis. First, the curated marker gene sets derived from prior literature or reported by the authors of the original studies were tested for enrichment within each cluster using Fisher’s exact test. *P*-values were adjusted with the Benjamini–Hochberg procedure. An adjusted *P*-value threshold of 0.05 was applied to determine significance. In addition, we performed classical pathway and GO term enrichment analysis using the EnrichR web tool [42, 43]. Only terms with an adjusted *P*-value ≤ .05 were considered significantly enriched. For visualization and interpretation, enrichment results were ranked by *P*-value, and only the top terms per cluster were reported.

### Benchmarking

To assess the specificity and performance of InCURA, we benchmarked its results against two alternative clustering strategies: (i) clustering on a randomized input matrix and (ii) co-expression-based clustering using WGCNA.

### Randomization of input matrix

For the random control, we first randomly shuffled the gene symbol column in the summarized output matrix from the FIMO scanning. This randomized motif hit table was then used to generate a TFBS count matrix of the same dimensions as the InCURA-derived gene-by-TF matrix by following the above-described workflow. The random matrix preserved the original shape and approximate value range of the motif count matrix but contained no biologically meaningful structure. It was then subjected to *k*-means clustering using the same number of clusters (*k = 4*) as applied in the InCURA workflow. The resulting clusters were then analyzed for enrichment of the same functional or marker gene signatures used to evaluate InCURA clusters.

### Weighted gene co-expression network analysis

For the co-expression benchmark, we used pyWGCNA for the bulk RNA-seq datasets and hdWGCNA for the single-cell pseudo-bulk datasets, applying the methods to the same set of DRGs that were submitted to InCURA [44, 45]. Normalized expression values were used to compute the pairwise correlation matrix. Soft-thresholding powers and dynamic tree-cutting parameters were selected to generate a number of gene modules that closely matched the number of InCURA clusters. Rather than strictly enforcing an identical number, we tolerated a ±1 module deviation to accommodate natural variability in WGCNA’s module detection. The resulting WGCNA modules were then evaluated using the same curated gene signatures and enrichment analysis applied to InCURA clusters.

### Implementation

We implemented InCURA as a Streamlit-based web application (Streamlit 2021, https://streamlit.io/). The web interface allows users to upload their input gene lists and either a list of expressed genes for TF filtering or TFs of interest. InCURA then performs filtering and the clustering workflow and returns downloadable gene modules in text format and TF enrichment results. For computational efficiency and to eliminate the need for local TFBS scanning, the application operates on a precomputed gene-by-TFBS matrix, which was generated by scanning promoter regions of all protein-coding genes in the reference genome with motifs of all available TFs (human or mouse, respectively). To validate the biological utility of this precomputed approach, we performed a proof-of-concept analysis using gene sets from curated databases. For the first benchmark, we compiled the union of genes from three Gene Ontology Biological Process terms (cytokine production, circadian clock, and cardiac muscle contraction) and used this combined set as input to InCURA. Clustering was performed using *k*-means, and the resulting modules were evaluated for overlap with the original GO terms. For a second benchmark focused on transcriptional regulation, we used the union of four hallmark regulatory target gene sets from the MSigDB database [46]: *Myc targets v1, IL2/STAT5 signaling, P53 pathway*, and *TGF-β signaling*. As in the first test, *k*-means clustering was applied to the TFBS profiles, and the clusters were evaluated based on their correspondence to the known gene sets. In cases where functional interpretation was ambiguous from GO enrichment alone, we performed cross-validation using Reactome pathway enrichment, which provided additional evidence for the regulatory identity of specific clusters.

## Results

### InCURA: integrative gene clustering on TFBS information

**Figure 1.**
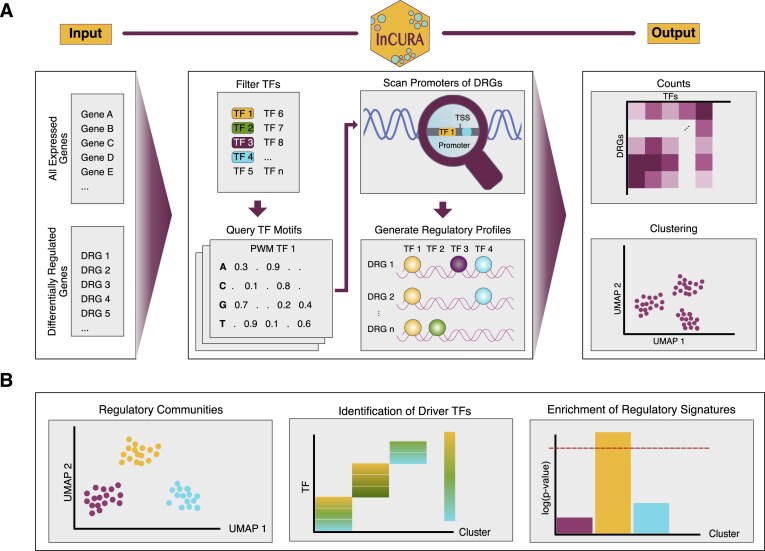
Overview of the InCURA workflow. (**A**) InCURA identifies regulatory gene modules by clustering DRGs based on shared TFBS patterns in their promoter regions. The input consists of DRGs and a list of all expressed genes that is used to identify expressed TFs. Promoter regions of DRGs are scanned for TF motifs using FIMO (MEME Suite), generating a gene-by-TFBS count matrix of motif occurrences. Unsupervised clustering (*k*-means) is applied to define gene modules with similar promoter-level regulatory control. (**B**) Possible downstream analyses include the detection of regulatory communities, the identification of driver TFs, and the enrichment of regulatory signatures.

To systematically uncover transcriptionally co-regulated gene modules, InCURA leverages TFBS information in promoter regions of DRGs and identifies clusters of genes that are likely driven by common upstream regulators (Fig. 1A). The input to the tool consists of two gene lists, derived from a single dataset, provided by the user: (i) a set of DRGs, representing the condition-relevant transcriptional response, and (ii) a list of all expressed genes in the dataset, from which the expressed TFs are extracted to restrict the search space to context-relevant regulators. The promoter regions of the DRGs are then scanned for binding motifs of the filtered TFs as defined by the PWMs from MotifDB using FIMO from the MEME Suite. By default, InCURA defines promoter regions as the interval from −2000 to +500 bp around the TSS, although this can be customized to suit specific use cases. The resulting TFBS predictions are stored in a gene-by-TFBS count matrix, where each entry reflects the number of predicted binding sites for a given TF in the promoter of a given gene and is treated as a regulatory profile for the analyzed gene. Genes are then clustered using unsupervised *k*-means clustering to define modules of genes that share similar predicted regulatory input. These modules can then serve as the basis for downstream analyses, including functional enrichment and identification of driver TFs (Fig. 1B). InCURA is available as a user-friendly web application (https://incura.streamlit.app/), and the full source code is accessible via GitHub (https://github.com/SinghalLab/incura_app) and archived on Zenodo (DOI: 10.5281/zenodo.15972184). For users requiring greater flexibility for customization, the workflow can be executed locally by cloning the repository.

### Application of InCURA to diverse transcriptomic datasets

To evaluate the versatility and biological relevance of InCURA, we applied the tool to four distinct case studies. These include two bulk RNA-seq datasets from both mouse and human, a mouse single-cell RNA-seq dataset, and published mouse differential expression information obtained from a web-based resource [29, 33, 34, 37]. Together, these use cases evaluate InCURA’s ability to recover biologically meaningful gene modules when applied to DEGs derived from diverse transcriptomic datasets. In each case, we show that the resulting clusters not only share predicted regulatory input but also align with known biological functions, pathways, or cell states. Additionally, we compared its output to two alternative clustering strategies: a random network and a co-expression-based approach using WGCNA. The primary aim was to evaluate whether the biologically meaningful gene modules identified by InCURA could also be recovered by methods that either ignore promoter-level regulatory information (WGCNA) or rely on randomized input. Therefore, we generated a random gene-by-TFBS matrix for each case study with the same dimensions as the InCURA-derived matrix and applied the same unsupervised clustering procedure. Further, we applied WGCNA (pyWGCNA for bulk data and hdWGCNA for single-cell data) to the same DEG sets that were used for the InCURA run.

### Case study 1: InCURA recovers effector T cell signatures from mouse bulk RNA-seq data

**Figure 2.**
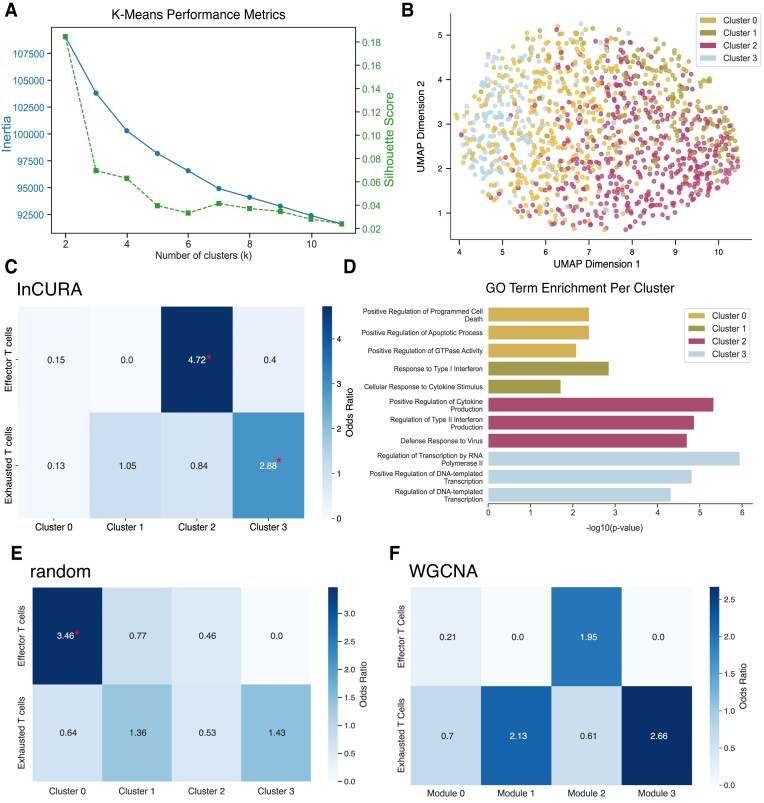
InCURA recovers effector T cell signature in TOX-knockout T cells during chronic viral infection. (**A**) Visualization of *k*-means performance of the input matrix measured by the inertia (elbow method) (left, blue) and the silhouette score (right, green). (**B**) UMAP visualization of gene clusters identified by InCURA, based on DEGs derived from the analysis of bulk RNA-seq data of TOX-knockout versus wild-type mouse T cells. (**C**) Odds ratios of the enrichment of marker genes for the effector T cell signature (top row) and the exhausted T cell signature (bottom row) in the InCURA clusters. Significance (*P*-value ≤ .05) is indicated by a red asterisk. (**D**) Top three enriched GO terms per cluster. (**E,F**) Odds ratios of the enrichment of marker genes for the effector T cell signature (top row) and the exhausted T cell signature (bottom row) in the random clusters (**E**) and WGCNA modules (**F**). Significance (Fisher exact test, Benjamini-Hochberg adj. *P*-value ≤ .05) is indicated by a red asterisk.

To assess whether InCURA can recover biologically meaningful gene modules from DEGs derived from bulk RNA-seq data, we applied the tool to a publicly available dataset of CD8⁺ T cells isolated from a TOX knockout mouse model with a well-characterized immune phenotype [29]. In this study, the authors demonstrated that the TF TOX is critical for the establishment and maintenance of exhausted T cells during chronic viral infection. Their transcriptomic analysis revealed that TOX knockout in T cells resulted in a loss of exhaustion-associated gene expression and the emergence of an effector T cell–like transcriptional signature. We hypothesized that these distinct regulatory programs could be recovered through InCURA clustering. Here, we computed DEGs between wild-type and TOX knockout T cell samples and submitted them to the InCURA pipeline, as described above. To determine the optimal number of clusters, we calculated both inertia and silhouette scores across a range of *k* values (2 to 12; Fig. 2A). Based on these performance metrics, we selected *k* = 4 for the main analysis, while also evaluating *k* = 3 and *k* = 5 for comparison (Supplementary Fig. S1). The resulting gene clusters (Fig. 2B) exhibited functional coherence, reflecting distinct cellular programs. Enrichment analysis of an independent effector T cell gene signature revealed significant overrepresentation in cluster 2 (Fig. 2C, top row). Furthermore, cluster 2 was also enriched for effector T cell-related pathways, including the positive regulation of cytokine production and the defense response to a viral infection (Fig. 2D). In contrast, the exhausted T cell signature was detected in cluster 3 (Fig. 2C, bottom row). The remaining clusters 0 and 1 showed enrichment for more general T cell functions. Notably, clustering on a random input matrix still captured the effector T cell signature (Fig. 2E), while the WGCNA modules captured neither of the tested gene programs (Fig. 2F). These results demonstrate that InCURA effectively identifies transcriptionally and functionally coherent gene modules, capturing biologically relevant regulatory patterns that extend beyond those resolved by conventional expression-based clustering or enrichment analysis, by considering shared TF–target gene interactions.

### Case study 2: InCURA identifies a specific B cell signature in response to systemic lupus erythematosus from human bulk RNA-seq data

**Figure 3.**
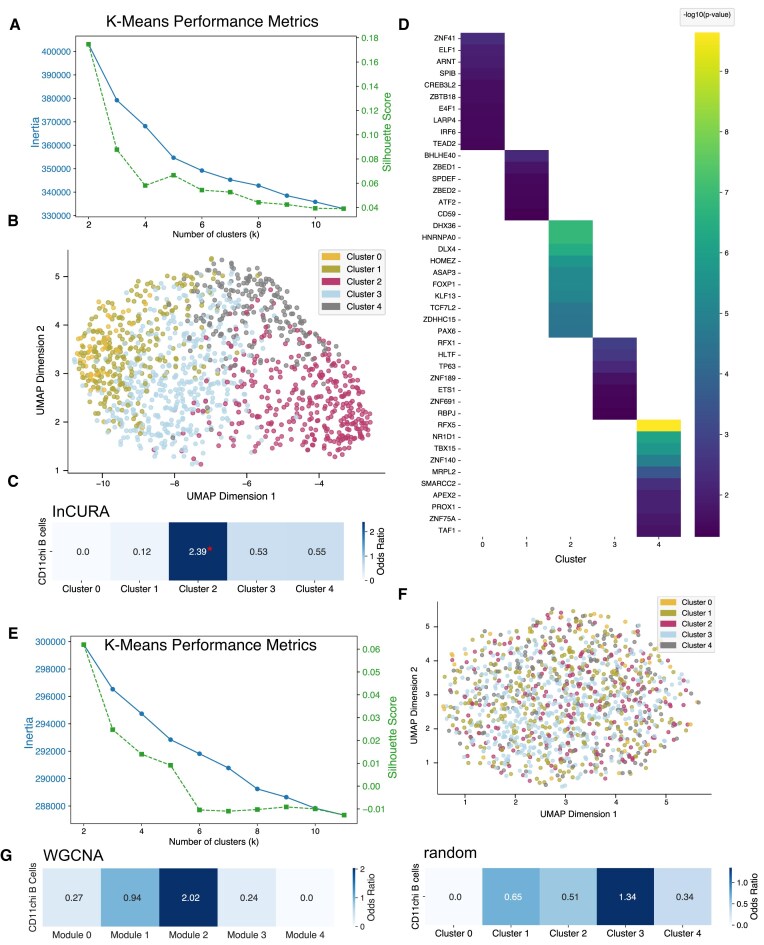
InCURA identifies SLE-specific B cell signatures. (**A**) Visualization of *k*-means performance of the InCURA input matrix measured by the inertia (elbow method) (left, blue) and the silhouette score (right, green). (**B**) UMAP visualization of gene clusters identified by InCURA, based on DEGs derived from the analysis of bulk RNA-seq data from B cells of SLE patients versus healthy individuals. (**C**) Odds ratios of the enrichment of marker genes for the CD11c⁺T-bet⁺ B cell signature in the InCURA clusters. (**D**) Top enriched TFs per cluster. (**E**) Visualization of *k*-means performance of the random input matrix measured by the inertia (elbow method) (left, blue) and the silhouette score (right, green). (**F**) UMAP visualization of gene clusters identified by random clustering. (**G**) Odds ratios of the enrichment of marker genes for the CD11c⁺T-bet⁺ B cell signature in the random clusters (right) and WGCNA modules (left). Significance (Fisher exact test, Benjamini–Hochberg adj. *P*-value ≤ .05) is indicated by a red asterisk.

Next, we assessed InCURA’s capacity to also recover biologically meaningful clusters in human data. Therefore, we applied InCURA to a public dataset of SLE patients [33]. SLE is a chronic autoimmune disease characterized by dysregulated B cell responses, yet the underlying molecular mechanisms remain not fully understood. This study identified a subset of CD11c⁺T-bet⁺ B cells with a distinct phenotype and transcriptome that is expanded in patients with SLE and may contribute to autoantibody production. To investigate whether InCURA can recover this disease-associated gene program, we performed differential gene expression analysis on bulk RNA-seq data derived from FACS-sorted B cells of SLE patients versus healthy individuals. Based on clustering performance metrics (inertia and silhouette score) (Fig. 3A), we selected *k* = 5 for downstream analysis. InCURA clustering revealed five distinct transcriptional modules (Fig. 3B), one of which (cluster 2) showed significant enrichment for the transcriptomic signature of the CD11c⁺T-bet⁺ B cell subset described in the original study (Fig. 3C). Further, TF enrichment analysis revealed that two known lupus-associated TFs, *KLF13* and *FOXP1*, were predicted as key regulators driving cluster 2 (Fig. 3D). Both TFs have been previously implicated in modulating immune activation and tolerance pathways relevant to lupus pathogenesis [47, 48]. Importantly, this disease-relevant gene signature was not recovered using either random clustering or a co-expression–based approach via WGCNA (Fig. 3E–G).

### Case study 3: InCURA recovers regulatory distinction between circadian and metabolic programs in hepatocytes

**Figure 4.**
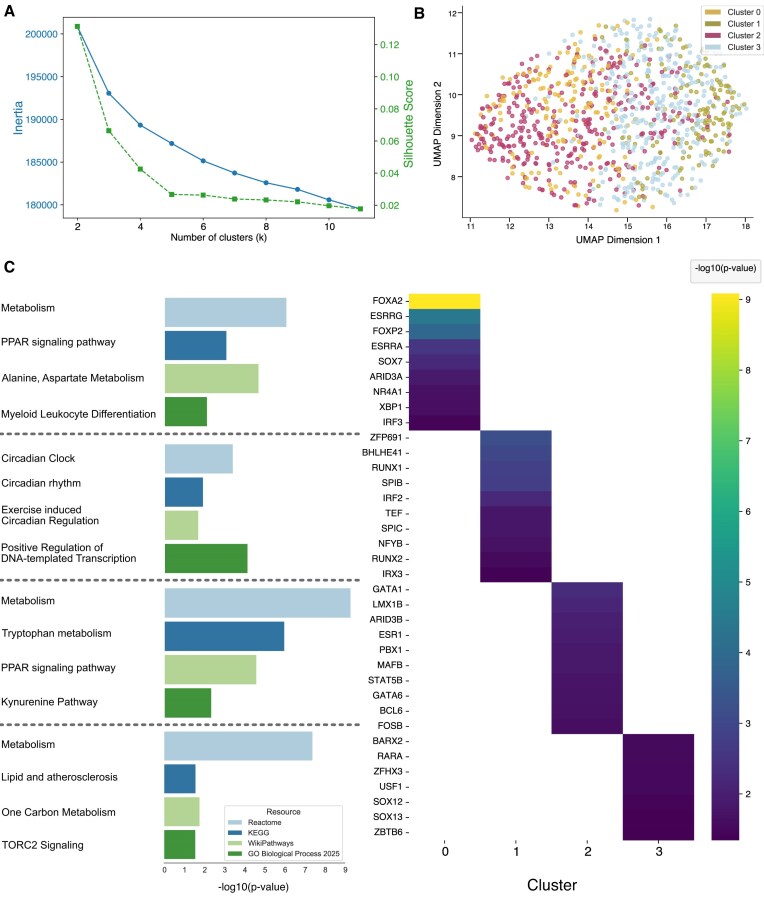
InCURA captures the distinction between circadian and metabolic regulatory programs. (**A**) Visualization of *k*-means performance of the InCURA input matrix measured by the inertia (elbow method) (left, blue) and the silhouette score (right, green). (**B**) UMAP visualization of gene clusters identified by InCURA, based on DEGs derived from the analysis of single-cell RNA-seq data from adult mouse hepatocytes of REV-ERBα/β double-knockout versus control liver tissues. (**C**) Enriched TFs driving each cluster (right) and corresponding enriched pathways as indicated by the −log_10_(*P*-values) based on four different resources (left): Reactome (light blue), KEGG (dark blue), WikiPathways (light green), and GO biological processes (dark green). From top to bottom: cluster 0–3.

To assess InCURA’s ability to resolve complex regulatory architecture in tissue-specific circadian perturbations, we analyzed single-cell RNA-seq data from hepatocytes isolated from adult mice with hepatocyte-specific double knockout of the core clock components REV-ERBα and REV-ERBβ [34]. This model disrupts the intrinsic circadian clock in hepatocytes while preserving systemic and non-hepatocytic rhythmic inputs. In addition to hepatocytes, the study also profiled non-parenchymal liver cells, including endothelial cells and Kupffer cells, highlighting the broader impact of clock disruption on liver physiology. For InCURA analysis, we focused specifically on the hepatocyte subset to identify modules of genes under shared regulatory control. We performed DEA between knockout and control hepatocytes and selected *k* = 4 for clustering based on inertia and silhouette score evaluations (Fig. 4A). The resulting InCURA clusters revealed distinct transcriptional programs, including one cluster that separated clearly from the others and was enriched for genes associated with circadian regulation, while the remaining clusters reflected metabolic pathways (Fig. 4B and C). This separation supports the functional relevance of the clusters and mirrors the dual disruption of rhythmic and metabolic processes described in the original study. Pathway enrichment analysis of InCURA clusters recovered several pathways highlighted in the original work, including PPAR signaling, one-carbon metabolism, and multiple amino acid metabolic pathways (Fig. 4C). Additionally, InCURA identified key TFs as potential upstream regulators of these clusters, such as *Esrra, Xbp1, Arid3b, Stat5b*, and *Bcl6*, all of which were also highlighted in the original study through cistrome-wide binding similarity analysis. Notably, neither random clustering nor WGCNA was able to resolve the regulatory separation between circadian and metabolic gene modules, further emphasizing the mechanistic specificity offered by InCURA’s motif-guided approach (Supplementary Fig. S2).

### Case study 4: InCURA identifies modules in DEGs derived from web-based resource

**Figure 5.**
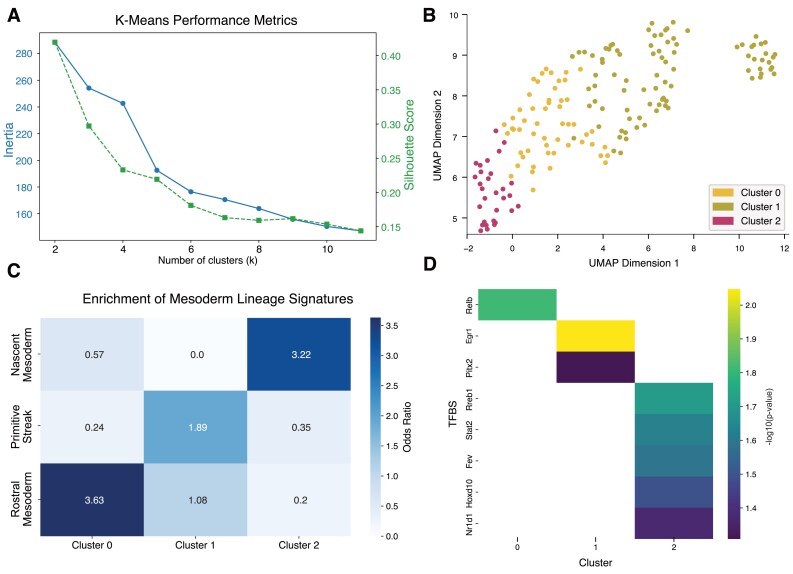
InCURA identifies developmental gene modules from a webtool-derived DEG list. (**A**) Visualization of *k*-means performance measured by the inertia (elbow method) (left, blue) and the silhouette score (right, green). (**B**) UMAP visualization of gene clusters identified by InCURA, based on DEGs extracted from a web-based tool accompanying a mouse gastrulation study. DEGs were derived from a comparison between mesodermal metacells representing early, intermediate, and late embryonic time points. (**C**) Odds ratios of the enrichment of marker genes for the different stages in the rostral mesoderm development. (**D**) Top-ranked TFs in each cluster.

Finally, we demonstrate that InCURA can also be applied to DEGs obtained from external databases, particularly in cases where raw transcriptomic data are not readily accessible. For this purpose, we selected a dataset investigating mouse gastrulation, in which single cells were computationally grouped into metacells and assigned embryonic age estimates [37]. The authors provided a web-based tool that enables DEA between any two metacells. We selected three mesoderm-lineage metacells, representing early, intermediate, and late embryonic time points, and extracted the resulting DEGs. Next, we used the union of the webtool’s DEA output between the early versus the intermediate metacell and the intermediate versus the late metacell as input for the InCURA workflow. We first evaluated the k-means performance metrics and found that the optimal number of clusters likely lies between 3 and 5 based on both inertia and silhouette score (Fig. 5A). Given the availability of three mesoderm-lineage stages in the dataset, we selected *k *= 3 for the downstream analysis with InCURA (Fig. 5B). To assess the biological relevance of the resulting clusters, we curated three gene signatures based on published marker genes, including those from the original study. These signatures corresponded to primitive streak cells, early nascent mesoderm, and rostral mesoderm. Although none of the three signatures reached statistical significance, the odds ratios indicated a strong enrichment trend across the three clusters, respectively (Fig. 5C). Additionally, the top-ranked TFs predicted to drive these clusters included regulators with known functions in gastrulation and mesoderm differentiation (Fig. 5D). For example, *Egr1* has been shown to play a critical role in early zebrafish mesoderm development, acting downstream of *Runx1* [49, 50]. Further, *Pitx2* and *Hoxd10* were identified as key drivers of clusters 1 and 2, respectively. While *Pitx2* is essential for primitive streak formation, *Hoxd10* is more likely to influence later developmental patterning [51, 52]. Together, these findings highlight the utility of InCURA in contexts where only summarized gene lists are available and illustrate its ability to recover biologically meaningful regulatory modules from minimal input.

### Application to non-transcriptomic features derived from chromatin accessibility data

**Figure 6.**
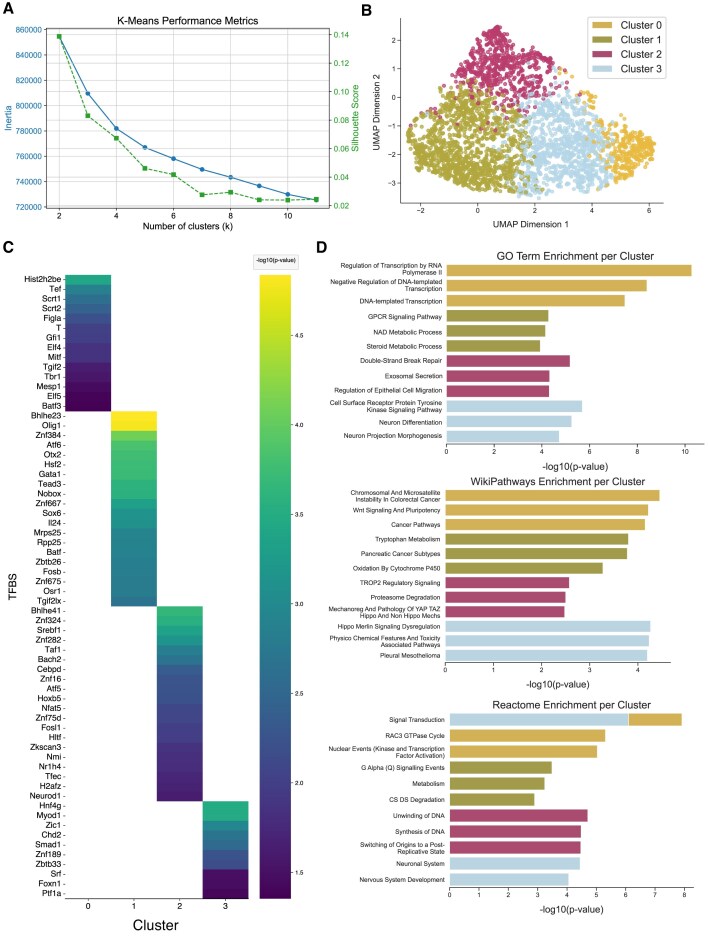
Application of InCURA to features derived from DARs of doxorubicin-resistant MCF7 breast cancer cells. (**A**) Visualization of *k*-means performance of the InCURA input matrix measured by the inertia (elbow method) (left, blue) and the silhouette score (right, green). (**B**) UMAP visualization of gene clusters identified by InCURA, based on genes linked to DARs derived from the analysis of bulk ATAC-seq data from doxorubicin-resistant versus doxorubicin-sensitive MCF7 breast cancer cells. (**C**) Top enriched TFs per cluster. (**D**) Top three enriched terms from enrichment analysis of GO biological processes, WikiPathways, and Reactome per cluster. The colors correspond to the legend in panel B.

To demonstrate the broader applicability of InCURA beyond transcriptomic data, we applied the tool to a feature set derived from a study identifying differentially accessible chromatin regions (DARs) with the Assay for Transposase-Accessible Chromatin using sequencing (ATAC-seq) [53] between doxorubicin-resistant and doxorubicin-sensitive MCF7 breast cancer cells [38]. In this setting, TF motif occurrences were quantified for the genes linked to these DARs by the original study (Fig. 6A and 6B). InCURA successfully identified coherent gene clusters enriched for cancer-related pathways, signaling pathways including YAP/TAZ and Hippo, and pathways associated with chromatin remodeling. The clusters were also enriched for functionally relevant TFs, such as members of the *AP-1, TEAD* and *FOX* TF families, which were consistent with the TFs highlighted in the original study (Fig. 6C and 6D).

### InCURA’s implementation as a user-friendly web tool enables accessible, regulation-centric clustering

**Figure 7.**
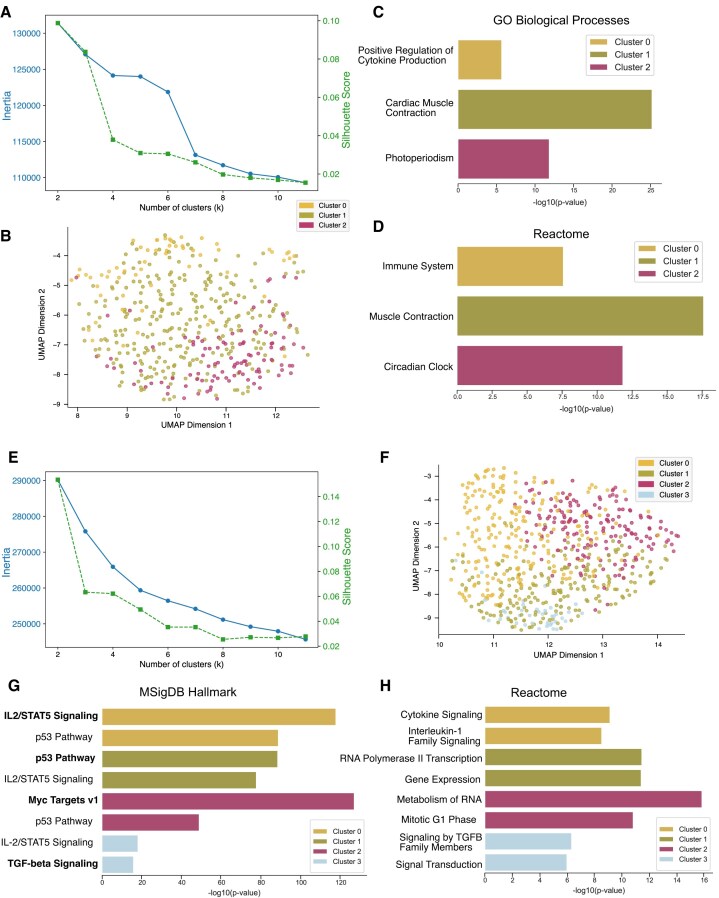
Validation with GO terms and MSigDB hallmark gene sets based on precomputed input matrix. (**A**) Visualization of *k*-means performance, based on GO term gene-by-TFBS count matrix, measured by the inertia (elbow method) (left, blue) and the silhouette score (right, green). (**B**) UMAP visualization of gene clusters identified by InCURA, based on the union of three different GO term gene sets (cytokine production, cardiac muscle contraction, and circadian clock). (**C**) GO term enrichment on identified InCURA clusters. (**D**) Cross-validation by enrichment of Reactome pathways. (**E**) Visualization of k-means performance, based on MSigDB gene sets gene-by-TFBS count matrix, measured by the inertia (elbow method) (left, blue) and the silhouette score (right, green). (**F**) UMAP visualization of gene clusters identified by InCURA, based on the union of four different MSigDB Hallmark gene sets (Myc Targets v1, p53 Pathway, IL2/STAT5 Signaling, and TGF-beta Signaling). (**G**) MSigDB Hallmark gene set enrichment on identified InCURA clusters. (**H**) Cross-validation by enrichment of Reactome pathways.

To support broad usability and reproducibility, we implemented InCURA as a user-friendly Streamlit-based web application. Users can upload their input gene lists, and the application returns gene clusters and TF prioritization. To ensure efficient runtime and platform independence, InCURA operates on a precomputed gene-by-TFBS matrix, enabling fast analysis without the need for local TFBS scanning. To validate that InCURA delivers biologically meaningful results even when using a precomputed genome-wide TFBS matrix, we performed a proof-of-concept analysis. As input, we used the union of genes from three Gene Ontology Biological Process terms: cytokine production, circadian clock, and cardiac muscle contraction. Despite combining diverse functional groups, InCURA successfully disentangled the input sets. Clustering based solely on TFBS features reproduced three distinct modules that corresponded directly to the original gene sets (Fig. 7A–D). We further tested the tool on a second, more regulation-focused set: the union of four hallmark regulatory target gene sets from MSigDB (*Myc targets v1, IL2/STAT5 signaling, P53 pathway*, and *TGF-β signaling*). Again, InCURA recovered clusters matching the original gene sets, demonstrating its ability to group genes based on regulatory input alone (Fig. 7E–H). However, in some cases, functional interpretation requires integration with multiple knowledge bases. For example, the cluster corresponding to *TGF-*β *signaling* did not emerge as the top enriched term in GO enrichment analysis but became clearly identifiable when cross-validated with Reactome pathway enrichment, confirming the TGF-β regulatory signature (Fig. 7G and H). These analyses highlight InCURA’s potential for unsupervised regulatory module discovery, even when individual motif scanning is disabled, and the gene-by-TFBS matrix is precomputed for the full genome. Its web-based implementation facilitates integration into a wide range of workflows, lowering the barrier to regulation-aware transcriptomic interpretation.

#### Discussion

InCURA offers a flexible and conceptually distinct approach to the functional interpretation of transcriptomic data by clustering genes based on predicted shared regulatory input, rather than on co-expression patterns or predefined gene annotations. By leveraging TFBS profiles in promoter regions, InCURA identifies gene modules that are likely co-regulated at the transcriptional level. This regulation-centric view provides a complementary layer of information to traditional differential expression and co-expression analyses, often revealing condition-specific modules that elude standard gene set enrichment or network-based clustering methods.

One of InCURA’s key strengths lies in the incorporation of regulatory information, while relying on minimal yet adaptable data input requirements: A list of DRGs and a list of all expressed genes from which context-specific TFs are filtered. This design makes it applicable across a wide range of experimental contexts, including scenarios where raw transcriptomic data are not readily available. However, it also means that InCURA is inherently reliant on the quality of upstream DEA. As with all DEG-based methods, results depend on the contrast being tested, sequencing depth, and statistical robustness [54, 55]. Importantly, functional signals identified through such analyses may reflect tissue composition or context-specific background noise rather than condition-specific regulation. This source bias complicates downstream interpretation, especially when using precurated pathway databases. In fact, preselection of DRGs can lead to significant pathway enrichments even in randomized data, undermining the specificity and interpretability of conventional enrichment approaches [56]. Nevertheless, analysis strategies like InCURA, which are independent of functional prior knowledge, allow inferring mechanistic insights while enabling a reliable biological interpretation.

For all datasets in this study, we used k-means clustering to group genes based on their TFBS profiles. Alternative data transformations, including log-transformation and truncated singular value decomposition (SVD), were explored to better meet k-means’ geometric assumptions. However, these transformations enhanced variation related to TF family composition (*R*² from a multivariate linear model = 0.6 with SVD versus 0.3 without SVD), which masked the variation associated with the biological condition of interest. Therefore, clustering was performed on the original TFBS count matrix. We selected *k* based on a combination of inertia, silhouette scores, and prior knowledge of the underlying biological structure. Our goal was to choose the smallest *k* that still captured meaningful regulatory patterns and avoid over-fragmentation into clusters that would be difficult to annotate or interpret biologically [57]. We acknowledge that this choice may miss finer regulatory distinctions. Moreover, in UMAP visualizations, clusters sometimes appear overlapping despite showing clear enrichment for distinct biological signatures, indicating that regulatory coherence may not always manifest as spatial separation in low-dimensional embeddings. To account for dataset-specific needs, the web implementation of InCURA allows users to adaptively choose *k* based on their data and research question.

Benchmarking results showed that InCURA outperforms both random clustering and WGCNA in identifying cell type- and context-specific gene modules. Notably, in one of the case studies, we observed a single statistically significant enrichment for one signature in the random clustering. This occurred in a dataset with a high number of DEGs and pronounced expression changes, which may increase the likelihood of coincidental enrichment. This underscores the need for multiple validation methods beyond statistical enrichment (e.g., TF enrichment, clustering performance metrics) when evaluating the biological relevance of the inferred modules. To further assess the specificity of InCURA’s clustering, we compared its performance to WGCNA. While WGCNA remains a powerful tool for detecting broad co-expression patterns, its reliance on global correlation structures across the transcriptome may obscure subtle but biologically meaningful regulatory programs. In contrast, InCURA clusters genes based on shared promoter architecture, which can highlight coordinated regulation even in the absence of strong expression correlation.

InCURA is also complementary to various tools designed to extract functional signatures from large datasets. For instance, GeneWeaver aggregates gene sets from diverse experimental sources and literature, enabling hypothesis-driven discovery research [58]. When used alongside InCURA, GeneWeaver can supply context-relevant gene sets, while InCURA adds a mechanistic regulatory layer through TF motif-based clustering. Similarly, iRegulon, which infers regulatory networks based on motif and ChIP-seq enrichment, aligns well with InCURA’s promoter-centric design [59]. While iRegulon is typically used downstream of co-expression networks to identify regulators, InCURA can be applied earlier, offering an unsupervised way to extract regulatory modules *de novo*. In comparison, gene regulatory network (GRN) inference frameworks, like SCENIC [40], provide deeper mechanistic insights by modeling the interactions between TFs and their target genes. Yet, GRN inference methods are typically computationally intensive, require substantial data preprocessing and parameter tuning, and often demand a higher level of computational expertise, limiting their accessibility to a broad user base, particularly the wet lab users. Additionally, they rely on different types of input data, making them less flexible than InCURA, which operates on simple gene lists. Thus, InCURA fills a methodological gap by providing a computationally lightweight, accessible alternative for extracting regulatory structure from transcriptomic data, while still being compatible with existing complex analytical frameworks. Together, these tools provide a versatile ecosystem for dissecting regulatory control in transcriptomic datasets.

Still, InCURA has limitations. In scenarios where regulatory control is diffuse or not sharply reflected in promoter motifs, the method may not recover coherent clusters as anticipated. This reflects a broader limitation of relying solely on promoter-level features in contexts where post-transcriptional regulation or distal enhancers might dominate. Moreover, the accuracy of TFBS-based clustering depends on the completeness and quality of the TF motif database. While tools like FIMO from the MEME suite enable comprehensive scanning, they usually produce a high proportion of false positive hits. Further, motif availability and resolution vary across species and TF families [60]. Additionally, InCURA depends on accurate TSS annotation, which can differ between genome builds and annotation sources, potentially affecting the definition of promoter regions and, in turn, the identification of regulatory motifs [61]. Finally, InCURA does not pre-split DRGs by up- and downregulation. While we thereby avoid imposing directional assumptions, we acknowledge that it represents a limitation, as the regulation direction is not explicitly modeled. However, if required by the user, the resulting gene clusters can be stratified by up- and downregulation during the downstream analysis process.

Despite these challenges, InCURA successfully identified biologically meaningful gene modules across diverse case studies, including bulk RNA-seq, single-cell transcriptomics, chromatin accessibility data, and web database-derived DEG sets. Taken together, InCURA contributes a valuable addition to the transcriptomics analysis toolbox. Due to its simple input requirements, InCURA can be readily integrated into any existing analysis workflow coordinated by frameworks such as BioConductor and scverse [62, 63, 64]. Its promoter-based clustering and motif-based feature space offer a robust alternative to current approaches. InCURA enables the extraction of biologically coherent gene modules in a regulation-aware manner that supports both discovery-driven and integrative analyses, especially in systems where co-expression and predefined annotations fall short.

## Supplementary Material

gkaf1377_Supplemental_Files

## Data Availability

The datasets used as case studies in this article are publicly available and can be downloaded from the corresponding sources, detailed in Supplementary Table S1. The full analysis pipeline is publicly available on Zenodo (https://doi.org/10.5281/zenodo.15972184) under the GNU General Public License v3.0. The customizable Snakemake-based version of InCURA can be downloaded from GitHub (https://github.com/SinghalLab/incura). The singularity image file needed to reproduce the environment is publicly available on Zenodo (https://doi.org/10.5281/zenodo.16031331).
