# Supplementary material for: InCURA: integrative gene clustering based on transcription factor binding sites
Source: Nucleic Acids Res. 2025 Dec 19;53(22):gkaf1377. doi: 10.1093/nar/gkaf1377 (PMC12715506; doi:10.1093/nar/gkaf1377)
Supplement: gkaf1377_Supplemental_Files [file gkaf1377_supplemental_files.zip › Rinck-et-al-Revised-Suppl-Materials.pdf]

InCURA: Integrative gene clustering based on transcription factor binding sites

Lorna Rinck[1,2], Ricardo O. Ramirez Flores[3], Julio Saez-Rodriguez[2,3], Mahak Singhal[1,4]

Supplementary Data

(Figure S1-S2 and Table 1)

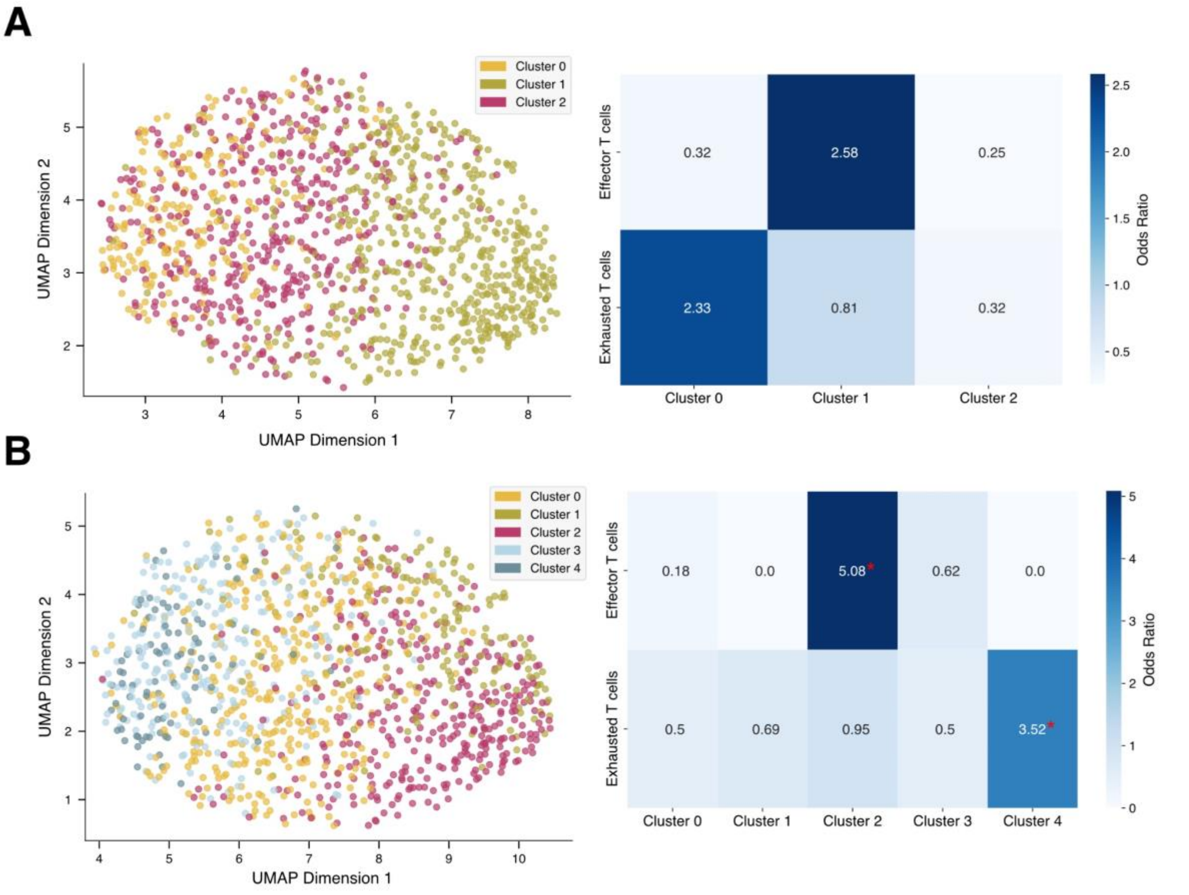

Supplementary Figure S1. Evaluation of different k values in T cell exhaustion case study. (**A**) InCURA clustering with k = 3. Left: UMAP visualization of the resulting gene clusters. Right: Corresponding odds ratios for enrichment of effector T cell and exhausted T cell signatures across clusters. (**B**) InCURA clustering with k = 5. Left: UMAP visualization of resulting gene clusters. Right: Corresponding odds ratios for enrichment of effector T cell or exhausted T cell signatures across clusters. A red asterisk indicates statistically significant enrichment (p-value ≤ 0.05).

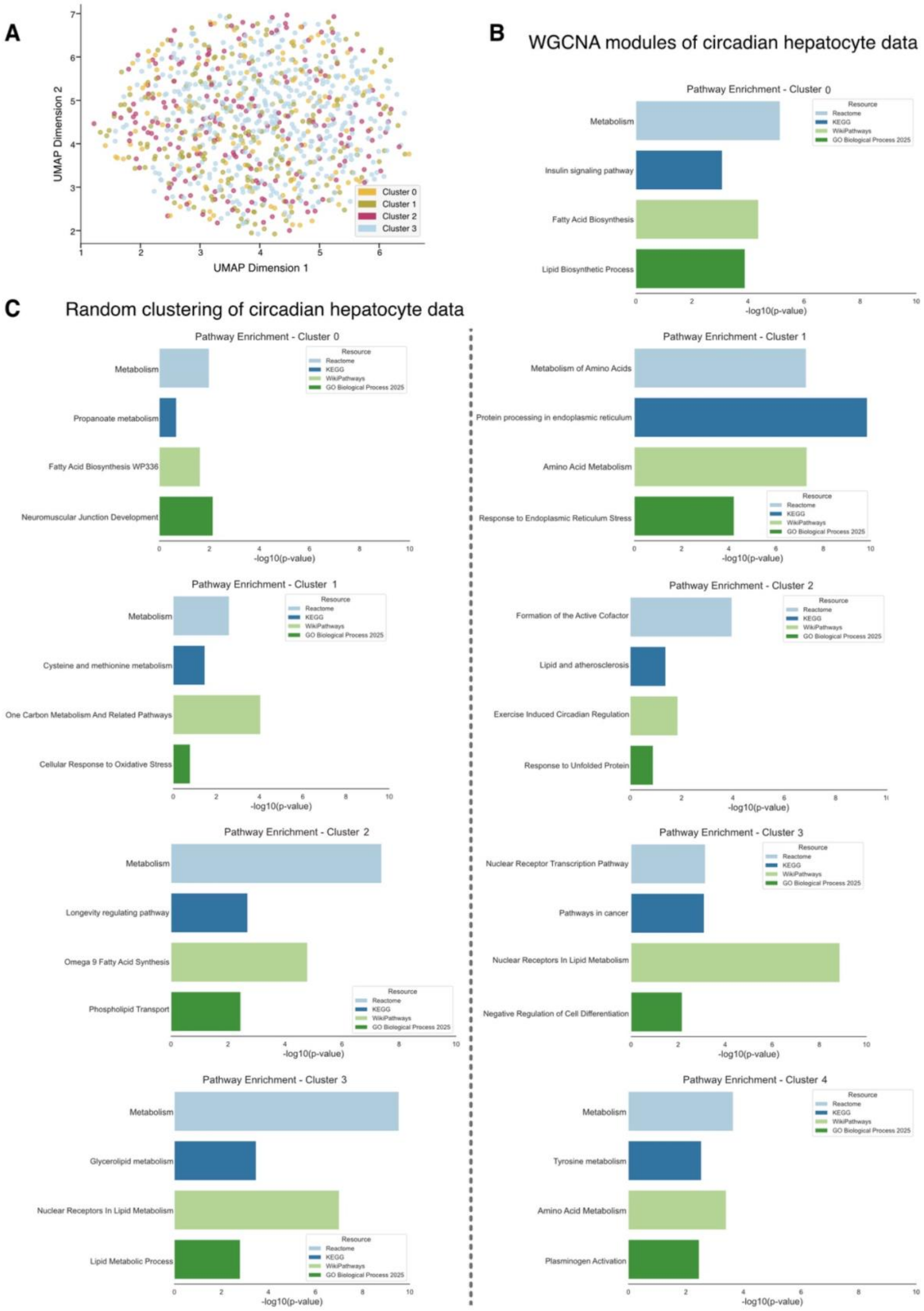
A
UMAP Dimension 2
UMAP Dimension 1
Cluster 0
Cluster 1
Cluster 2
Cluster 3
B
WGCNA modules of circadian hepatocyte data
Pathway Enrichment - Cluster 0
Metabolism
Insulin signaling pathway
Fatty Acid Biosynthesis
Lipid Biosynthetic Process
Resource
Reactome
KEGG
WikiPathways
GO Biological Process 2025
-log10(p-value)
C
Random clustering of circadian hepatocyte data
Pathway Enrichment - Cluster 0
Metabolism
Propanoate metabolism
Fatty Acid Biosynthesis WP336
Neuromuscular Junction Development
Pathway Enrichment - Cluster 1
Metabolism of Amino Acids
Protein processing in endoplasmic reticulum
Amino Acid Metabolism
Response to Endoplasmic Reticulum Stress
Pathway Enrichment - Cluster 1
Metabolism
Cysteine and methionine metabolism
One Carbon Metabolism And Related Pathways
Cellular Response to Oxidative Stress
Pathway Enrichment - Cluster 2
Formation of the Active Cofactor
Lipid and atherosclerosis
Exercise Induced Circadian Regulation
Response to Unfolded Protein
Pathway Enrichment - Cluster 2
Metabolism
Longevity regulating pathway
Omega 9 Fatty Acid Synthesis
Phospholipid Transport
Pathway Enrichment - Cluster 3
Nuclear Receptor Transcription Pathway
Pathways in cancer
Nuclear Receptors In Lipid Metabolism
Negative Regulation of Cell Differentiation
Pathway Enrichment - Cluster 3
Metabolism
Glycerolipid metabolism
Nuclear Receptors In Lipid Metabolism
Lipid Metabolic Process
Pathway Enrichment - Cluster 4
Metabolism
Tyrosine metabolism
Amino Acid Metabolism
Plasminogen Activation

Supplementary Figure S2. Random clustering and hdWGCNA cannot capture distinction between circadian and metabolic regulatory programs. (**A**) UMAP visualization of gene clusters identified by random, based on DEGs derived from the analysis of single-cell RNA-seq data from adult mouse hepatocytes of REV-ERBα/β double-knockout vs. control liver tissues. (**B**) Enriched pathways in hdWGCNA modules as indicated by the -log10(p-values) based on four different resources: Reactome (light blue), KEGG (dark blue), WikiPathways (light green) and GO Biological Processes (dark green). From top to bottom: Module/cluster 0 - 4. (**C**) Enriched pathways in random clusters as indicated by the -log10(p-values) based on four different resources: Reactome (light blue), KEGG (dark blue), WikiPathways (light green) and GO Biological Processes (dark green). From top to bottom: Cluster 0 - 3.

Supplementary Table 1. External data used in case studies

**Case Study**	**Data Type**	**Species**	**Accession**	**Authors**	**Year**	**Link**
T cell exhaustion	Bulk RNA-seq	mouse	GSE132987	Khan *et al.*	2019	-
SLE B cells	Bulk-cell RNA-seq	human	GSE110999	Wang *et al.*	2018	-
Circadian double knockout	Single-cell RNA-seq	mouse	GSE143528	Guan *et al.*	2021	-
Mouse gastrulation	DEGs only	mouse	GSE169210	Mittenzweig *et al.*	2021	https://apps.tanaylab.com/MCV/embflow/
MCF7 breast cancer cells	Genes linked to DARs	human	GSE174152	Wang *et al.*	2021	-
